# Characteristic spatial and frequency distribution of mutations in *SCN1A*

**DOI:** 10.1186/s42494-024-00178-z

**Published:** 2024-11-11

**Authors:** Mengwen Zhang, Jing Guo, Bin Li, Kang Liu, Jiayuan Zhao, Jiayuan Zhang, Xuqing Lin, Bin Tang, Jie Wang, Weiping Liao, Na He

**Affiliations:** 1 Department of Neurology, Institute of Neuroscience, Key Laboratory of Neurogenetics and Channelopathies of Guangdong Province and the Ministry of Education of China, The Second Affiliated Hospital, Guangzhou Medical University, Guangzhou, 510260 China; 2 Department of Neurology, The Guangdong 999 Brain Hospital, Guangzhou, 510510 China; 3 Guangzhou Medical University, Guangzhou, 511436 China

**Keywords:** Epilepsy, *SCN1A*, CpG dinucleotides, Mutation hotspot, Spatial and frequency distribution

## Abstract

**Background:**

*SCN1A* is the most well-recognized and commonly mutated gene related to epilepsy. This study analyzed the characteristic spatial and frequency distributions of *SCN1A* mutations, aiming to provide important insight into the mutagenesis etiopathology of *SCN1A*-associated epilepsy.

**Methods:**

Epilepsy-associated *SCN1A* variants were retrieved from the *SCN1A* mutation database, the HGMD database, and literature reviews. The base substitutions, mutation frequencies in CpG dinucleotides, and spatial distributions of mutations in terms of exons and structural domains were analyzed.

**Results:**

A total of 2621 *SCN1A* variants were identified in 5106 unrelated cases. The most common type was missense mutation, followed by frameshift mutations and splice site mutations. Among the missense mutations, transitions within CpG dinucleotides were much more recurrently identified than transitions within non-CpG dinucleotides, and the most common type was the G > A transition. Among the nonsense mutations, the most predominant type of single-base substitution was the C > T transition, among which 75.3% (235/312) were within CpG sites. The most common “hotspot” codons for missense mutations were codons 101, 946, and 1783; while for nonsense mutations it was codon 712. One-base deletion or insertion was the most common type of frameshift mutation, causing protein truncation. The three most common frameshift mutations were c.5536_5539delAAAC, c.4554dupA, and c.5010_5013delGTTT. Splice mutations were the most frequently identified in exon 4 with a hotspot site c.602 + 1G > A. The spatial distribution of missense mutations showed that exons 22 and 4 had the highest mutation density (111 and 84 mutations per 100 bp, respectively), and exon 12 had the lowest mutation density, with 4 mutations per 100 bp. Further distribution analysis of the protein domains revealed that missense mutations were more common in the pore region and voltage sensor (231 mutations per 100 amino acids, respectively), and the protein truncation mutations were distributed evenly among the domains.

**Conclusions:**

*SCN1A* mutations tend to cluster at distinct sites, depending on the characteristic CpG dinucleotides, exons, and functional domains. Higher mutation density in particular regions, such as exon 22 and exon 4, offers promising targets for therapeutic genetic interventions.

## Background

The *SCN1A* gene (MIM *182389), located on human chromosome 2q24 and containing 26 exons, encodes the pore-forming α subunit of the neuronal voltage-gated sodium channel Nav1.1. The Nav1.1 channel is a large single-chain polypeptide consisting of 2009 amino acids that forms two terminal regions (N-terminal and C-terminal regions) and four homologous domains, designated as DI to DIV. Each of the four homologous domains has six transmembrane helical segments: five hydrophobic segments (S1, S2, S3, S5, and S6) and one positively charged segment (S4). The S4 segment and its link regions serve as voltage sensors that are important for voltage sensing. The S5 and S6 segments and their extracellular connecting regions form the pore region of the channel [1–3].

The *SCN1A* gene stands as the foremost and most prevalent gene associated with epilepsy. Variants in *SCN1A* give rise to a wide range of epilepsy syndromes, ranging from mild familial febrile seizures (FEB) [4], and genetic epilepsy with febrile seizures plus (GEFS +) [5], to severe developmental and epileptic encephalopathy (DEE) or Dravet syndrome (DS) [6–8]. In addition to epilepsy, *SCN1A* variants are also associated with other disorders, including familial hemiplegic migraine (FHM) and autism spectrum disorders (ASD) [9, 10].

Currently, *SCN1A* mutations have been reported to be distributed widely across the protein, which include missense mutations, nonsense mutations, frameshifts, splice site variants, small in-frame insertions/deletions, and large insertions/deletions. Among them, missense mutations and truncating mutations that cause protein truncations through nonsense, frameshift, or invariant splice site mutations are the most commonly observed alterations [11, 12]. Clinically, phenotype severity is closely related to the location and type of mutation. Generally, truncation mutations and missense mutations in the pore region are usually associated with severe epilepsies such as DS, whereas missense variants outside the pore region are associated with mild epilepsies [3, 13]. Understanding the type of base substitutions and spatial distribution of *SCN1A* mutations provides essential information for assisting in precision clinical management and treatment.

In the present study, we systematically reviewed *SCN1A* mutations associated with epilepsy and analyzed the spatial and frequency distributions, aiming to provide an important insight into the mutagenesis etiopathology of *SCN1A*-associated epilepsy.

## Methods

We systematically reviewed all *SCN1A* mutations associated with epilepsy from the *SCN1A* mutation database (http://scn1a.caae.org.cn/) [3] and the Human Gene Mutation Database (http://www.hgmd.cf.ac.uk/index.php, version: HGMD Professional) till December 2023, which collected *SCN1A* mutations published since 2000 [14]. The results from the two databases were cross-checked to avoid duplicate recruitment. The relevant literature was searched and double-checked in the PubMed database. All *SCN1A* mutations were annotated based on the transcript NM_001165963.4.

In this study, large deletions or large insertions were defined as those involving one or more exons, and those affecting fewer than one exon (one or several base pairs) were classified as small deletions or insertions involving frameshift mutations, in-frame mutations or splicing mutations. Mutations, including intronic single base pair substitutions, deletions, or insertions within invariant 5′GT or 3′AG dinucleotides and deletions that spanned the coding sequence and the intronic invariant dinucleotides, were classified as splicing mutations. The cDNA sequence was scanned for CpG dinucleotides. Altogether, 105 CpG dinucleotides were located throughout the whole cDNA sequence. These CpG dinucleotides can be classified into three forms in the coding sequence: CGN, NCG, and NNC GNN, according to the location of the CpG dinucleotides in a codon.

Missense, nonsense, and frameshift mutations were mapped across the Nav1.1 protein. To analyze whether the distribution of these mutations occurred randomly or clustered in special regions, we divided the entire protein into the following six domains: (1) voltage sensor: the S4 segments and their linkers; (2) pore region: regions from the S5 to S6 segments and their neighboring distal portion (approximately 26–35 amino acids; http://www.uniprot.org/uniprot/P35498); (3) segments S1-S3: regions in the four internal homologous domains except the “voltage sensors” and “pore regions”; (4) D-linkers: linkers between the four homologous domains (DI–DII, DII–DIII, and DIII–DIV); (5) N-terminus: the amino terminal domain; and (6) C-terminus: the carboxy terminal domain.

### Statistical analysis

An affected family was considered as a single case in the data analysis. Considering that a given mutation was recurrently reported in unrelated affected families, to avoid confusion, we use "variant" to represent different changes in cDNA (such as base substitutions or different changes at the same cDNA site), and "case" to refer to unrelated individuals or families. The mutation frequencies, hotspot identification, and mutation densities were based on the number of cases. A hotspot mutation refers to a variant that is repeatedly reported in a large number of cases. For exons, mutation densities were calculated as the number of mutations per 100 base pairs; for domains, mutation densities were calculated as the number of mutations per 100 amino acids to adjust for the different sizes.

A Chi-square test was performed to determine the difference in the domain distributions between missense mutations and protein truncation mutations. SPSS 27.0 software was used for statistical analyses. A *P*-value < 0.05 was considered statistically significant.

## Results

A total of 2621 *SCN1A* variants were identified in 5106 unrelated cases, among which 1582 variants were reported only once, and 1039 variants were recurrently reported in 3524 cases. Among all the cases, the most common type was missense mutations (2743/5106, 53.7%), followed by frameshift mutations (921/5106, 18.0%), nonsense mutations (688/5106, 13.5%), and splice site mutations (483/5106, 9.5%). Small insertions/deletions (79/5106, 1.5%), large insertions/deletions (170/5106, 3.3%), complex rearrangements (13/5106, 0.3%), and regulatory substitutions (1/5106, 0.02%) were also observed in the *SCN1A* gene (Table 1).

**Table 1:** Distribution of *SCN1A* mutations by mutation types

Mutation type	Variants	Cases
Translation initiation	5 (0.19%)	8 (0.16%)
Missense		
Transition within CpG dinucleotides	139 (5.30%)	547 (10.71%)
Within non-CpG dinucleotides	1157 (44.14%)	2196 (43.01%)
Nonsense		
Transition within CpG dinucleotides	29 (1.11%)	262 (5.13%)
Within non-CpG dinucleotides	217 (8.28%)	426 (8.34%)
Frameshift truncation		
Frameshift deletion	421 (16.06%)	645 (12.63%)
Frameshift insertion	157 (5.99%)	237 (4.64%)
Frameshift indel	34 (1.30%)	39 (0.76%)
Splice donor site	146 (5.57%)	325 (6.37%)
Splice acceptor site	89 (3.40%)	158 (3.09%)
In-frame deletion	41 (1.56%)	68 (1.33%)
In-frame insertion	9 (0.34%)	11 (0.22%)
Large deletion	144 (5.49%)	149 (2.92%)
Large insertion	20 (0.76%)	21 (0.41%)
Complex rearrangements	12 (0.46%)	13 (0.25%)
Regulatory substitutions	1 (0.04%)	1 (0.02%)
Total	2621 (100.0%)	5106 (100.0%)

### Distribution of missense mutations

**Fig. 1:**
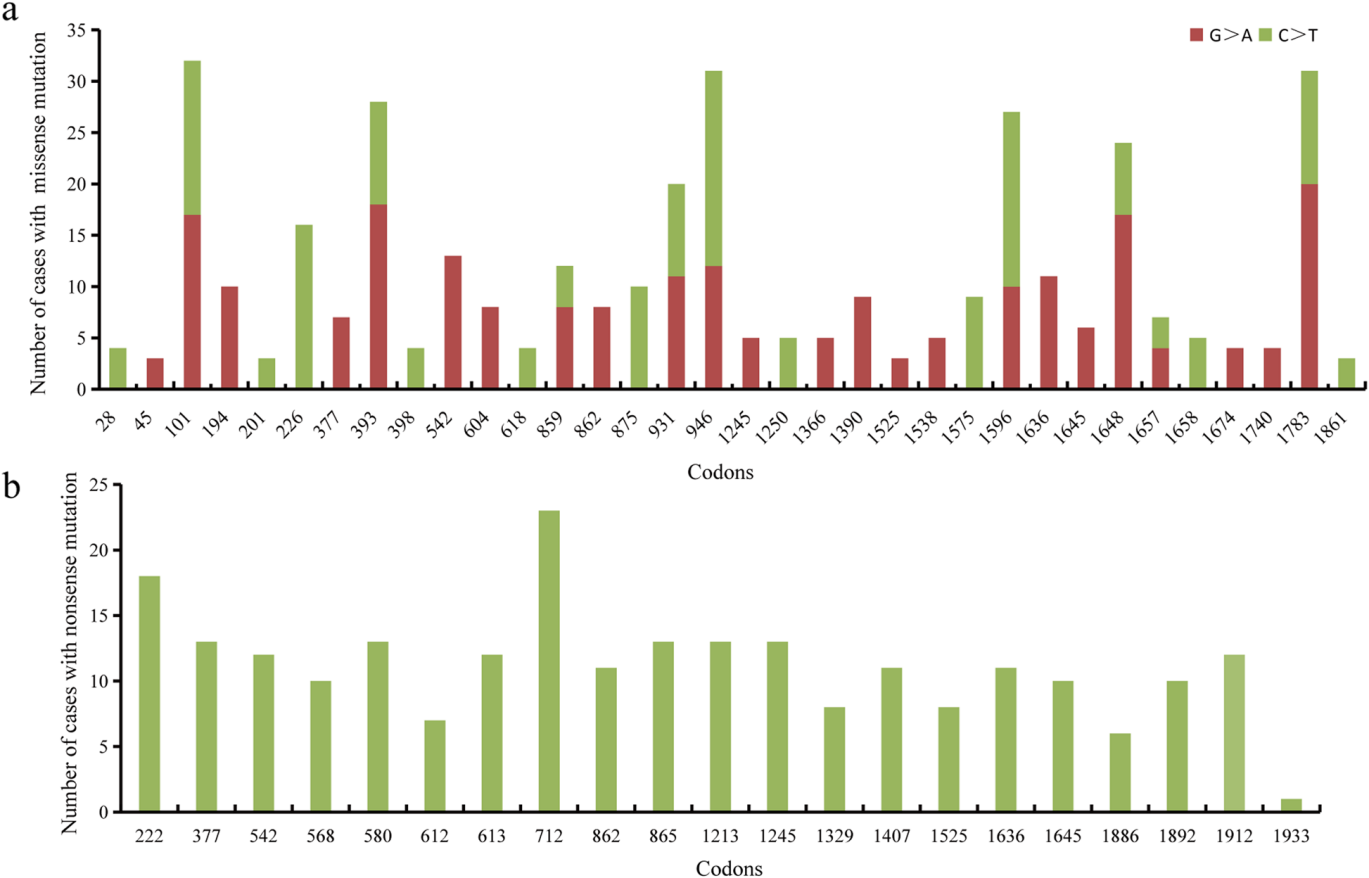
**a** Distribution of missense mutations (≥ 3 cases) in CpG codons. **b** Distribution of nonsense mutations in CGA codons

Among the missense mutations, transitions within CpG dinucleotides were more recurrently identified than transitions within non-CpG dinucleotides (139 variants recurrently in 547 cases vs 1157 variants recurrently in 2196 cases). In terms of base transitions (Table 2), G > A transitions (521/2743, 19.0%) were the most common type, followed by T > C (431/2743, 15.71%) and C > T transitions (413/2743, 15.1%). A total of 547 cases with missense mutations, which were clustered within 85 codons with CpG dinucleotides, accounted for 81.0% of all CpG dinucleotides in the entire cDNA sequence. The distribution of these missense mutations in codons is presented in Fig. 1a. The most recurrent missense mutation site was codon 101 (32 times: 15 times for c.301C > T and 17 times for c.302G > A), followed by codon 1783 (31 times: 11 times for c.2836C > T and 20 times for c.2837G > A) and codon 946 (31 times: 19 times for c.2836C > T and 12 times for c.2837G > A). Codon 1783 (GCG), which is flanked by a 5′ cytosine residue and contains 2 CpG dinucleotides (NNC GCG), potentially represents the second most recurrent missense mutation site.

**Table 2:** Base substitutions of *SCN1A* point mutations and their distribution in different mutation types

Base substitution	Total	Missense	Nonsense	Splice
C > T				
Within CpG dinucleotides	413 (10.69%)	178 (6.49%)	235 (34.16%)	0 (0.00%)
Within non-CpG dinucleotides	321 (8.28%)	235 (8.57%)	77 (11.19%)	9 (2.03%)
C > A	192 (4.95%)	132 (4.81%)	51 (7.41%)	9 (2.03%)
C > G	172 (4.44%)	145 (5.29%)	21 (3.05%)	6 (1.35%)
G > A				
Within CpG dinucleotides	271 (6.99%)	242 (8.82%)	0 (0.00%)	29 (6.53%)
Within non-CpG dinucleotides	496 (12.80%)	279 (10.17%)	100 (14.53%)	117 (26.35%)
G > C	231 (5.96%)	186 (6.78%)	0 (0.00%)	45 (10.14%)
G > T	326 (8.41%)	166 (6.05%)	101 (14.68%)	59 (13.29%)
A > G	277 (7.15%)	236 (8.60%)	0 (0.00%)	41 (9.23%)
A > C	117 (3.02%)	96 (3.50%)	0 (0.00%)	21 (4.73%)
A > T	152 (3.92%)	88 (3.21%)	33 (4.80%)	31 (6.98%)
T > C	466 (12.03%)	431 (15.71%)	0 (0.00%)	35 (7.88%)
T > A	239 (6.17%)	165 (6.02%)	51 (7.41%)	23 (5.18%)
T > G	202 (5.21%)	164 (5.98%)	19 (2.76%)	19 (4.28%)
Total	3875 (100.00%)	2743 (100.00%)	688 (100.00%)	444 (100.00%)

### Distribution of nonsense mutations

Among the nonsense mutations, transitions within CpG dinucleotides were highly mutated and repetitive, with 29 nonsenses recurrently reported in 262 cases. The most predominant type of single-base substitution was the C > T transition (312/688, 45.3%), among which 75.3% (235/312) were within CpG sites (Table 2). All 235 cases with nonsense mutations within CpG dinucleotides had mutations in CGA codons (where codon CGA was changed to the termination codon TGA), which converted C > T transitions by methylation-mediated deamination of 5-methylcytosine. The CGA > TGA transitions were distributed among 21 CGA codons (Fig. 1b), with codon 712 being the most recurrent site, with 23 cases, while codon 1933 was reported only once.

### Distribution of frameshift mutations

**Fig. 2:**
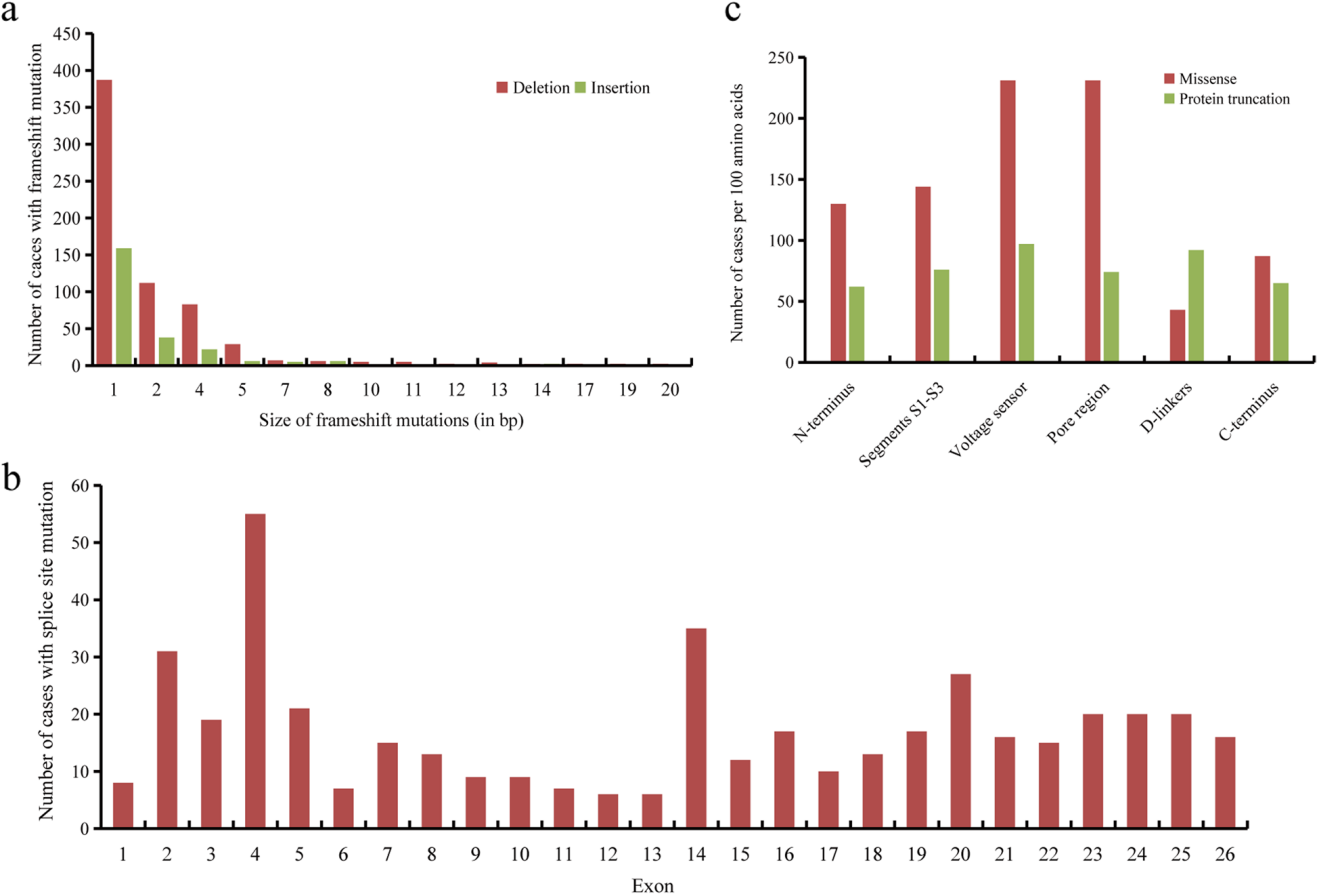
**a** Size distributions of frameshift mutations in *SCN1A*. **b** Spatial distributions of *SCN1A* splice site mutations by exon. The location of splice site mutations is categorized by the adjacent exon. **c** Distribution of *SCN1A* mutation types by domains. Protein truncations include nonsense mutations and frameshift mutations. χ.^2^ test for comparison between missense and protein truncations, *P* < 0.001

A total of 612 frameshift variants have been reported in 921 unrelated cases, among which 438 variants were reported only once, and 174 variants were recurrently identified in 483 cases (483/921, 52.4%). We then explored the size distributions of frameshift mutations in *SCN1A*. There were three times more base deletions than base insertions (645 vs 237, Table 1), causing frameshift truncation of *SCN1A*. The size of the base insertions/deletions ranged from one base pair to 20 base pairs, with one base deletion or insertion being the most common type (Fig. 2a). Notably, the 12-base pair deletion (c.644_655del12) was reported once due to the formation of a termination codon (TGA) by the 5' T and the 3' GA (GCATtgagaacattcaGAGTT), consequently leading to protein truncation. The three most common frameshift mutations are as follows: the four-base pair AAAC deletion in the cDNA sequence CCACAACCaaacAAACTCCA (c.5536_5539delAAAC, recurrently reported 16 times), the one-base pair A insertion in the cDNA sequence AAAaAAA (c.4554dupA, repetitively 8 times), and the four-base pair GTTT deletion in the cDNA sequence GCGTTgtttAAC (c.5010_5013delGTTT, repetitively 7 times).

### Distribution of splice site mutations

There were twofold more splice donor site mutations than splice acceptor site mutations (325/158, Table 1). The majority of the splice site mutations (401/483, 83%) were single-base substitutions within 5 bp of the intron-exon boundary, 75.8% of which (304/401) were within invariant 5′GT or 3′AG dinucleotides (i.e., canonical ± 1 or 2 splice sites). Ten splicing mutations were within the deep intron regions, about 2000 bp far apart from the intron-exon boundary, among which nine were within or near a cryptic evolutionarily conserved “poison” exon 20N within intron 20. Splicing mutations were most often identified in exon 4 (Fig. 2b), owing to a hypermutable CpG dinucleotide that was reported 29 times (c.602 + 1G > A) [15–17]. Exons 14, 2, and 20 also exhibited relatively higher frequencies of splicing mutations. No other discernable spatial or frequency patterns were observed for the other exons.

### Mutation densities by exon

The mutation densities of *SCN1A* missense, nonsense, and frameshift mutations within the 26 coding regions are shown in Table 3. To adjust for the different sizes of *SCN1A* exons, the mutation densities were calculated as the number of mutations per 100 base pairs. The average mutation densities were 45 mutations/100 bp, 11 mutations/100 bp, and 15 mutations/100 bp for missense, nonsense, and frameshift mutations, respectively. Among missense mutations, the highest density was in exon 22 (111 mutations/100 bp), which encodes the pore region, followed by exon 4 (84 mutations/100 bp). Exon 12, which encodes the DI–DII linker, had the lowest mutation density, with 4 mutations/100 bp. The mutation densities in exons showed no obvious differences for nonsense and frameshift mutations.

**Table 3:** Mutation densities by exon within *SCN1A* coding regions

Exon	Length (bp)	Missense		Nonsense		Frameshift	
		No	per 100bp	No	per 100bp	No	per100bp
1	264	81	31	21	8	27	10
2	119	97	82	8	7	24	20
3	90	30	33	6	7	12	13
4	129	109	84	16	12	14	11
5	92	71	77	21	23	9	10
6	270	120	44	17	6	34	13
7	64	42	66	4	6	13	20
8	142	115	81	33	23	34	24
9	207	113	55	36	17	35	17
10	285	30	11	36	13	57	20
11	381	53	14	54	14	40	10
12	133	5	4	25	19	14	11
13	239	51	21	21	9	19	8
14	174	62	36	20	11	36	21
15	357	296	83	39	11	52	15
16	483	73	15	38	8	66	14
17	121	16	13	8	7	35	29
18	155	42	27	40	26	19	12
19	174	83	48	40	23	37	21
20	123	62	50	12	10	34	28
21	282	193	68	31	11	48	17
22	54	60	111	6	11	8	15
23	138	83	60	13	9	19	14
24	105	57	54	30	29	46	44
25	271	141	52	14	5	40	15
26	1178	658	56	99	8	149	13
Total	6030	2743	45	688	11	921	15

### Mutation densities by protein domain

To illustrate the mutation densities in the protein domains, we classified each mutation into six domains: the voltage sensor, the pore region, segments S1-S3, D-linkers, N-terminus, and C-terminus. To avoid bias caused by the different sizes of domains, the mutation densities were calculated as the number of mutations per 100 amino acids (aa). There were significant differences in the domain distribution between missense mutations and protein truncation mutations (*P* < 0.001). The mutation density of missense mutations in the pore region and the voltage sensor was greater (both 231 mutations per 100 aa, respectively), while the density in the other domains was 43–144 mutations per 100 aa; protein truncation mutations are evenly distributed across the six domains. (Fig. 2c).

## Discussion

*SCN1A* is one of the most commonly mutated genes associated with epilepsy with a typical autosomal dominant inheritance pattern. The majority of the mutations occur de novo in the affected children, while a small proportion are inherited from mosaic affected or unaffected parents [18]. *SCN1A* mutations have different functional impacts, including loss of function (LOF), partial loss of function, decreased excitability, increased excitability, and gain of function (GOF) [3, 18]. Generally, truncation mutations and missense mutations with loss of function in the pore region and the voltage sensor are associated with severe epilepsies such as DS [3]. In the present study, we systematically analyzed the spatial and frequency distributions of mutations in *SCN1A*. Although *SCN1A* mutations are distributed widely across the protein, their distribution displays nonrandom patterns with hypermutability of CpG dinucleotides and uneven distributions of missense mutations. *SCN1A* mutations tend to be clustered at distinct sites, depending on the characteristic CpG dinucleotides, exons, and functional domains.

Transitions within CpG dinucleotides are highly mutated and recurrently reported; 139 missense transitions within CpG dinucleotides have been recurrently reported in 547 cases, and 29 nonsense transitions within CpG dinucleotides have been recurrently reported in 262 cases, indicating that these CpG dinucleotides may be mutation hotspots in the *SCN1A* gene. At the DNA base pair level, this nonrandom pattern is basically due to the hypermutability of CpG dinucleotides (Table 2). DNA methylation at the cytosine residue of CpG dinucleotides produces 5-methylcytosine, which results in a C > T and corresponding G > A transition on the complementary DNA strand by deamination. These cytosine nucleotides are particularly susceptible to mutations during meiosis and early embryogenesis due to methylation [19]. The reason why methylated CpG dinucleotides have a high rate of transition is that both exogenous and endogenous mutagens preferentially target methylated CpG dinucleotides over their unmethylated counterparts [20–22]. Approximately 60–90% of cytosine residues in CpG dinucleotides in the human genome are methylated, while unmethylated dinucleotides are primarily clustered in promoter regions of housekeeping genes [23]. CpG dinucleotide methylation has important implications for the etiology of genetic disease, as suggested by the fact that 20–50% of point mutations in different genes result from transitions within CpG dinucleotides [24–27].

Interestingly, these hypermutable CpG transitions in nonsense mutations preferentially occur at CGA codons, where C > T transitions change arginine codons (CGA) to stop codons (TGA). CGA is the only codon for which deamination of a CpG dinucleotide generates a stop codon. Although arginine codons 222 and 712 were the most frequent mutant codons (Fig. 1b), there was no evidence showing that the spatial distribution of the arginine codons influenced the mutation frequency: codon 222 was located in DIS4, whereas codon 712 was distributed in D-linkers. Furthermore, while the 3′ CGA codon 1933 has only 1 mutation, there is no evidence showing that 5′ CGA codons are preferentially mutated with respect to 3′ CGA codons (Fig. 1b). However, the most frequent mutant codons resulting in missense mutations were located in the pore region (codons 393, 946 and 1783) and the voltage sensor (codon 1648) (Fig. 1a), suggesting that the structural location influences the frequency of missense mutations.

The uneven spatial pattern of *SCN1A* missense mutations by exon revealed that exon 22 had the highest mutation density (111 mutations per 100 bp), followed by exons 4, 15, 2, 8 and 5, most of which were the exons encoding segments S4–S6. Further spatial distribution by structural domain showed that missense mutations had higher mutation densities in functional structures such as the voltage sensor and the pore region than on basic structures (e.g., D-linker, S1–S3). One of the explanations may be the clinical ascertainment bias. Mutations in the voltage sensor and pore region can cause severe phenotypes (e.g., DS) that are receiving increased clinical attention [28], while mutations in the “insulating” region can cause a milder phenotype that may be ignored and do not undergo *SCN1A* mutation screening. In contrast, protein truncating mutations, including nonsense mutations, frameshift mutations, and splice site mutations that lead to the absence or aberrant synthesis of the gene product, are associated with phenotypes of similar clinical severity. Therefore, these variants had no obvious hotspots in terms of exons or structural domains.

The structure-function relationships of the Nav1.1 channel protein may be another reason for the nonrandom pattern of missense mutations. The S5–S6 segments form the pore region and play an important role in ion selectivity; S4 serves as a voltage sensor and is crucial for action potential initiation [1, 2, 29]. Single amino acid substitutions within these segments can alter ion specificity and excitability, thereby causing seizures. *SCN1A* mutations in pore regions are commonly associated with LOF, whereas mutations in regions of channel inactivation (S4–5 and D3–4 linkers) are often associated with GOF [13, 28, 30]. Functional property analyses on 437 sodium channel-variants demonstrated that pore-forming regions were frequently associated with LOF variants, inactivation domains were associated with GOF, and voltage-sensing regions were associated with variants with both LOF and GOF [30]. The evidence showed that the types and/or locations of *SCN1A* mutations can predict the functional property of mutations to some extent. Previously studies have indicated that *SCN1A* mutations do not exclusively affect inhibitory neurons [31, 32], but also excitatory neurons [12]. Deficits in the channel Nav1.1 in the inhibitory neurons are indisputable for the pathophysiology of severe phenotype, such as DS [33]. The excitatory neuronal networks were affected differently depending on the type of *SCN1A* mutation; for instance, the missense versus nonsense mutations and pore domain versus voltage-sensing domain influence the excitatory network phenotype. However, these effects on excitatory neuronal networks did not correlate with the clinical severity [12].

The present study revealed that there were three times more frameshift deletions than frameshift insertions, which is similar to findings in other human genes [34]. The three most common frameshift mutations are c.5536_5539delAAAC, c.4554dupA, and c.5010_5013delGTTT, which were repeatedly reported 16, 8, and 7 times, respectively. All three most common frameshift mutations occurred in a sequence context of short nucleotide repeats. These repetitive sequences are prone to slipped-strand mispairing (SSM) during DNA replication or repair, leading to insertion or deletion errors. Specifically, when DNA polymerase or other repair enzymes slip over these repetitive sequences, mismatches can occur. These mismatches may result in the insertion or deletion of one or more nucleotides, thereby causing frameshift mutations [35, 36].

Previous studies have identified several disease-associated mutations in intron 20 that are within an extremely highly conserved mammalian sequence, the “poison” exon (PE), 20N, whose inclusion is predicted to lead to transcript degradation [37]. Aantisense oligonucleotide can prevent the inclusion of the PE 20N in human *SCN1A*, increasing the production of the *SCN1A* transcript and NaV1.1 expression [38, 39]. In the present study, splicing mutations were most often identified in exon 4, suggesting a targeted therapeutic direction for clinical intervention in this specific region.

Taken together, the results of this study revealed that the distribution of *SCN1A* mutations is nonrandom. Hotspots, such as CpG dinucleotides or an exon encoding a key part of the biological function of the protein, were highly susceptible to mutation. We emphasize that mutation detection strategies should focus on CpG dinucleotides in a critical domain of the protein, especially CGA (arginine) codons, which can create termination codons terminated by C > T transitions. Since such nonsense mutations are, on average, more deleterious than missense mutations, they may reasonably be expected to result in phenotypes that are more readily detectable clinically. Higher mutation density in particular regions, such as exon 22 and exon 4, offers promising avenues for targeted therapeutic interventions, such as precision medicine approaches and gene editing strategies.

## Conclusions

This study revealed the characteristic spatial and frequency distribution of mutations in *SCN1A*, which demonstrated a nonrandom pattern with mutations clustered at distinct sites, depending on the characteristic CpG dinucleotides, exons, and functional domains.

## Data Availability

The original contributions presented in the study are included in the article, further inquiries can be directed to the corresponding authors.

## Abbreviations

aa: amino acids
DS: Dravet syndrome
GOF: Gain of function
LOF: Loss of function
PE: Poison exon
